# Selenium Polysaccharide SPMP-2a from* Pleurotus geesteranus* Alleviates H_2_O_2_-Induced Oxidative Damage in HaCaT Cells

**DOI:** 10.1155/2017/4940384

**Published:** 2017-02-15

**Authors:** Yujun Sun, Cheng Zhou, Shoucheng Huang, Changjun Jiang

**Affiliations:** ^1^College of Life Sciences, Anhui Agricultural University, Hefei 230036, China; ^2^School of Life Science, Anhui Science and Technology University, Fengyang 233100, China; ^3^State Key Laboratory of Tea Plant Biology and Utilization, Anhui Agricultural University, Hefei 230036, China

## Abstract

Selenium- (Se-) enriched polysaccharide SPMP-2a was extracted and purified from* Pleurotus geesteranus*. SPMP-2a is a white flocculent polysaccharide and soluble in water, with a molecular weight of 3.32 × 10^4^ Da. Fourier transform infrared spectroscopy spectral analysis indicated that it belongs to an acid Se polysaccharide with *α*-D-glucopyranoside bond. The effects of Se polysaccharide SPMP-2a in* P. geesteranus* against hydrogen peroxide- (H_2_O_2_-) induced oxidative damage in human keratinocytes (HaCaT) cells were evaluated further. Reduced cell viability and elevated apoptotic rates in H_2_O_2_-treated HaCaT cells were proven by MTT and flow cytometry assays. Hoechst 33342 staining revealed chromatin condensations in the nuclei of HaCaT cells. However, with the addition of SPMP-2a, cell viability improved, nuclear condensation declined, and cell apoptotic rates dropped significantly. Ultrastructural observation consistently revealed that treatments with SPMP-2a reduced the number of swollen and vacuolar mitochondria in the H_2_O_2_-treated cells compared with the controls. Furthermore, SPMP-2a increased the superoxide dismutase (SOD) and catalase (CAT) activities and reduced reactive oxygen species (ROS) content. Western blot analysis showed that SPMP-2a treatment effectively increased B-cell lymphoma 2 (Bcl-2) protein expression. Therefore, SPMP-2a could improve cellular antioxidant enzyme activities, reduce ROS levels, and increase Bcl-2 protein expression levels, thereby reducing cell apoptosis and protecting HaCaT cells from H_2_O_2_-induced oxidative damage.

## 1. Introduction

In living organisms, reactive oxygen species (ROS) such as superoxide anion free radical (O^2−∙^), hydroxyl free radical (^*∙*^OH), and hydrogen peroxide (H_2_O_2_) can be generated [1]. Excessive ROS production exceeds its ROS-scavenging ability, which causes inflammation, aging, and even cancer [2]. ROS-mediated oxidative stress has been shown to be correlated with the occurrence of various diseases (cardiovascular diseases, e.g., hypertension, dyslipidemia, and obesity) and cell apoptosis [3]. Previous studies have indicated that H_2_O_2_ can lead to direct oxidation of membrane lipid and proteins [4]. Furthermore, it can pass through the cell membrane and produce strong activity of free radicals such as ^*∙*^OH, thereby inducing cell apoptosis through various metabolic pathways [5].

Increasing evidence has indicated that fungal polysaccharides can scavenge free radicals, inhibit lipid peroxidation, and delay aging [6, 7]. Edible fungi polysaccharides are known as biological response modifiers [8]. Wang et al. [9] reported that polysaccharides from* Cordyceps sinensis* enhance the activities of glutathione peroxidase (GSH-Px) and superoxide dismutase (SOD), thereby eliminating the accumulation of ROS, including O^2−∙^ and ^∙^OH. Gao et al. [10] showed that exopolysaccharides from* Russula vinosa* have strong in vitro antioxidant activity to scavenge DPPH free radical, O^2−∙^, and ^∙^OH. Moreover, selenium (Se) is one of the essential trace elements for animals and humans and is an important component of GSH-Px, which is an antioxidant in red blood cells [11]. Se constitutes the active centers of several oxidases [12], promotes peroxide decomposition, and protects cell membrane structures [13]. Inorganic Se is the primary form of Se in nature but is difficult for animals and humans to absorb [14]. In addition, inorganic Se has greater toxicity than organic Se, and excessive intake is detrimental to the animal body [15]. Studies found that edible mushrooms are capable of accumulating Se [16]. Edible mushrooms link inorganic Se with polysaccharides, which convert inorganic Se into organic Se polysaccharide. Organic Se polysaccharide has both polysaccharides and Se, which the human body can easily absorb [17].

The mushroom* Pleurotus geesteranus* belongs to* Dikarya subkingdom*,* Basidiomycota phylum*, Agaricomycotina subphylum, Agaricomycetes class, Agaricales order, Pleurotaceae family, and* Pleurotus* genus [18]. It is a popular edible mushroom with a unique flavor and smooth taste. Polysaccharides from* P. geesteranus* have strong antioxidant [19], blood lipid lowering [20], and antitumor properties [21]. However, information about the Se-combining polysaccharide of* P. geesteranus* is scarce. In our previous study, polysaccharides extracted from* P. geesteranus* exhibited higher superoxide radical- and hydroxyl radical-scavenging activities in a dose-dependent manner [22]. The present study uses MTT assay to examine cell viability, Hoechst 33342 fluorescence staining to show apoptotic cell morphology, flow cytometry to detect apoptotic rates of HaCaT cells, and Western blot analysis to investigate its protective effects and the underlying mechanisms of SPMP-2a on H_2_O_2_-induced oxidative damage in human keratinocytes (HaCaT). Results showed that Se-combining polysaccharide from* P. geesteranus* (SPMP-2a) reduced oxidative stress-induced cell death. This study provided important evidence that SPMP-2a has great potential to alleviate oxidative stress and cell damage.

## 2. Materials and Methods

### 2.1. Bacterial Strains and Cell Lines

*Pleurotus geesteranus* (GIM5.217) was purchased from the Institute of Microbial Culture Collection in Guangdong and identified by rDNA-ITS sequence analysis (GenBank number KY417089). HaCaT cell lines were obtained from Shengbo Biopharmaceutical Co. (Shanghai, China). DEAE-Sepharose Fast Flow and Superdex-200 were purchased from Amersham Biosciences Co. (Uppsala, Sweden). Sodium selenite was acquired from Sigma Chemical Co. (St. Louis, MO, USA).

### 2.2. Preparation of* P. geesteranus* Se Polysaccharide

*P. geesteranus* was inoculated on potato dextrose agar (PDA) culture medium for activation (long-time preserved strain before culturing). After two rounds of culture, the seed culture solution, approximately 10% (v/v) of the final culture solution, was added to a fermentation tank (FUS-50L, Guoqiang Biochemical Engineering Equipment Co., Ltd., Shanghai, China) with the addition of 20 ug/mL selenite sodium. Liquid culture was performed for 7 d at 180 rpm and 25°C with 0.9 vvm (air volume/culture volume/min), and centrifugal separation was used to collect the mycelia.

Se-enriched mycelium was made into powder and subsequently added to distilled water. After being treated at 70°C for 3 hours (h), the extract was concentrated and precipitated by adding threefold volume of 95% ethanol (v/v) and keeping it at 4°C for 24 h. After centrifugation at 4800 rpm for 10 min, the supernatant was collected and freeze-dried. The Se-enriched polysaccharide from mycelia of* P. geesteranus* (SPMP) was obtained.

SPMP was dissolved in distilled water and then fractionated by DEAE-Sepharose Fast Flow (2.6 cm × 50 cm) with a discontinuous gradient elution of distilled water and 1 mol/L NaCl at 1.0 mL/min [23]. The elution profile was made by the phenol-sulfuric acid assay, and two elution peaks, SPMP-1 and SPMP-2, were visualized. SPMP-2 was further applied to Superdex-200 (1.6 cm × 60 cm) with the AKTA™ Purifier 10 system and eluted with distilled water at 1.0 mL/min flow rate. The elution profile showed three elution peaks, namely, SPMP-2a, SPMP-2b, and SPMP-2c.

### 2.3. Determination of Purity and Molecular Weight of SPMP-2a

The purity and molecular weight (MW) of SPMP-2a were determined by high performance liquid chromatography (HPLC) on an Agilent 1200 system (Agilent Technologies, Palo Alto, CA, USA). A total of 20 *μ*L polysaccharide solution (10 mg/mL) was injected and eluted with double distilled water at 35°C at 1.0 mL/min flow rate [26]. A series of concentration gradients of dextran was used to draw the standard curve and make the regression equation, which was used to calculate the MW of SPMP-2a.

### 2.4. FTIR Spectral Analysis of SPMP-2a

The functional groups of SPMP-2a were detected by Fourier transform infrared (FTIR) spectrometer. SPMP-2a (1 mg) was ground into powder and mixed with 100 mg KBr powder. The mixture was then pressed into pellets for FTIR spectra measurement in the 400–4000 cm^−1^ frequency range [24].

### 2.5. Effects of SPMP-2a and H_2_O_2_ on HaCaT Cell Viability

The HaCaT cells at their logarithmic growth phases were seeded at densities of 1 × 10^5^ cells/mL into a 96-well cell culture plate. A 100 *μ*L medium containing various concentrations of SPMP-2a (0, 100, 200, 300, 400, or 600 *μ*g/mL) or H_2_O_2_ (0, 50, 100, 200, 400, 600, or 800 *μ*mol/L) was added to the cell plate after 24 h. After a 20-hour incubation period, the cell medium was aspirated and 20 *μ*L 5 mg/mL MTT solution was added and cultured for 4 h. The supernatant was discarded, and 150 *μ*L DMSO was added to dissolve the precipitate. The optical density (OD) value was measured at 490 nm, and the cell survival rates were calculated. Each treatment was repeated five times. The cell survival rates were calculated by the following formula: (%) = (OD treated group/OD blank) × 100%.

### 2.6. Effects of SPMP-2a against H_2_O_2_-Induced Cell Death in HaCaT Cells

The experiments were divided into the following groups: the control group, the model group, and the SPMP-2a treatment groups (100, 200, and 300 *μ*g/mL), plus five replicates for each group. The HaCaT cells at their logarithmic growth phases were seeded at densities of 1 × 10^5^ cells/mL into a 96-well cell culture plate with a 100 *μ*L medium. After a 24-hour incubation period, the 100 *μ*L medium containing various concentrations of SPMP-2a was added and incubated for 20 h. Except for the control group, 100 *μ*L of 200 *μ*mol/L H_2_O_2_ was added in the other four groups and incubated for another 6 h before cell viability was calculated.

### 2.7. Cell Staining and Cell Apoptosis Detection

The HaCaT cells were seeded into culture dishes at densities of 1 × 10^5^ cells/mL. The cell medium was aspirated and washed with phosphate buffer saline (PBS) after SPMP-2a treatment. The Hoechst 33342 dye was diluted to 10 *μ*g/mL in PBS. Cells were stained with Hoechst 33342 at room temperature for 30 min and rinsed with PBS to remove the dye. Cell morphology was examined using confocal laser scanning microscopy (FV1000, Olympus, Japan).

The HaCaT was seeded in 6-well plates at 2 × 10^5^ cells/well density. Cell damage was induced by H_2_O_2_ and alleviated by polysaccharides. The cells were digested with trypsin and collected by centrifuging at 2000 rpm for 5 min. The cells were washed with PBS followed by centrifuging at 2000 rpm for 5 min. A total number of 1–5 × 10^5^ cells were collected and resuspended in a 100 *μ*L 1x binding buffer. A total of 5 *μ*L Annexin V-FITC and 5 *μ*L PI of staining solution were added to the cell suspension, mixed gently, and incubated in the dark at room temperature for 10 min. Then, 400 *μ*L 1x binding buffer was added to the mixed cells. Apoptosis was detected by flow cytometry (FACSAria, American BD Company).

### 2.8. Ultrastructural Observation

Cell samples were collected and fixed in 2.5% glutaraldehyde at room temperature for 2 h. Then, the samples were rinsed three times with PBS (0.1 M, pH 7.2) and fixed in 1.0% osmium tetroxide for 1 h, followed by rinsing with PBS. Subsequently, the samples were dehydrated, embedded, and cut into thin sections (approximately 70 nm). Ultrathin sections were observed using transmission electron microscopy. At least 10 individual cells were observed. Each sample was sectioned three times, and representative photographs were selected.

### 2.9. SOD and CAT Activity and ROS Content Measurement

Cells were digested, collected, and lysed with a RIPA buffer. Cell lysate was centrifuged at 12000 rpm/min at 4°C for 5 minutes. The supernatants were collected and tested according to the SOD and CAT kit instructions. DCFH-DA was used as fluorescent probe according to DCFH-DA kit instructions. Flow cytometry was used to detect ROS content in the samples, with 488 nm excitation wavelength of 530 nm emission wavelength.

### 2.10. Western Blot Analysis

All proteins were extracted and subjected to SDS-PAGE. After being transferred to a membrane and blotted with 5% skim milk, the primary antibodies (anti-Bcl-2, 1 : 1000, and *α*-tubulin 1 : 500) were incubated with membrane overnight at 4°C. A horseradish peroxidase conjugate antibody (1 : 10000) washed with PBS was incubated thereafter with the membrane at room temperature for 1 h. The enhanced chemiluminescence method was used for X-ray film exposure, development, and fixing. The primary antibodies were purchased from Cell Signaling Technologies (Danvers, MA), and the secondary antibodies were purchased from Abcam (ab8229).

### 2.11. Data Analysis

SPSS 18.0 software was used for statistical analysis. The results were denoted as the mean ± standard deviation. We used five replicates for each data to minimize measurement error. *t*-test determined the significant difference. *P* < 0.05 (or 0.01) was the suggested significant difference.

## 3. Results

### 3.1. Isolation and Purification of Se-Enriched Polysaccharide

**Figure 1:**
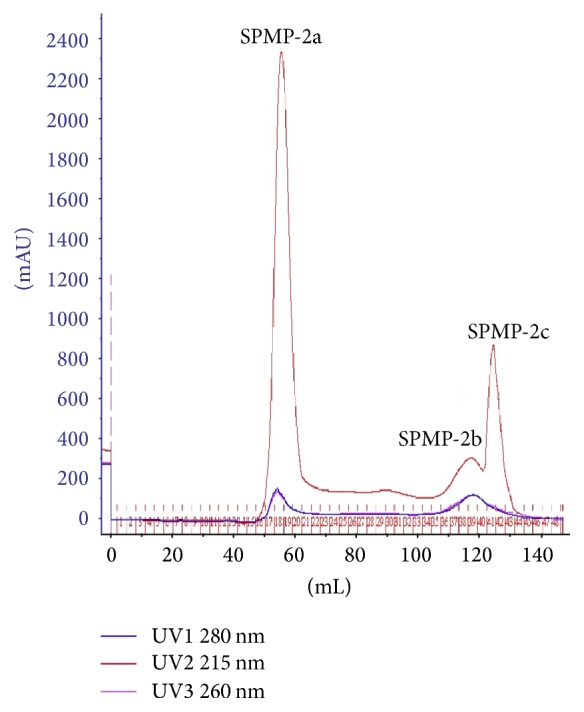
Graphical representation reveals the separation and purification of SPMP-2 by AKTA Superdex-200 system. Three polysaccharide fractions (SPMP-2a, SPMP-2b, and SPMP-2c) were shown.

SPMP was isolated and purified by DEAE-Sepharose Fast Flow column chromatography, in which the neutral polysaccharide (SPMP-1) was eluted by distilled water and the polyanion polysaccharide (SPMP-2) was eluted by sodium chloride. SPMP-2 was further separated by AKTA Superdex-200 gel column chromatography, and three kinds of polysaccharide fractions (SPMP-2a, SPMP-2b, and SPMP-2c) were obtained (Figure 1). SPMP-2a was the highest component, accounting for 71.6% of the total SPMP. SPMP-2a is a white flocculent polysaccharide that is soluble in water, but not in ethanol, acetone, or other organic solvents. SPMP-2b and SPMP-2c could not be separated completely; thus, SPMP-2a was selected for further studies.

### 3.2. Purity and MW of SPMP-2a

**Figure 2:**
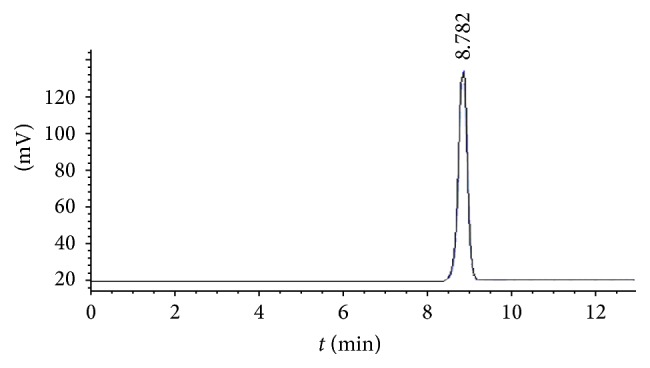
HPLC result of SPMP-2a. The retention time (8.782 min) was shown.

The HPLC result of SPMP-2a showed a single long and narrow symmetrical peak, which suggested that SPMP-2a was highly pure and homogeneous (Figure 2). According to the retention time (8.782 min) and the standard curve equation (log⁡Mw = 8.9006*t* − 0.4987, *R*^2^ = 0.9988), the MW of SPMP-2a was estimated to be 3.32 × 10^4^ Da.

### 3.3. FTIR Spectral Analysis of SPMP-2a

**Figure 3:**
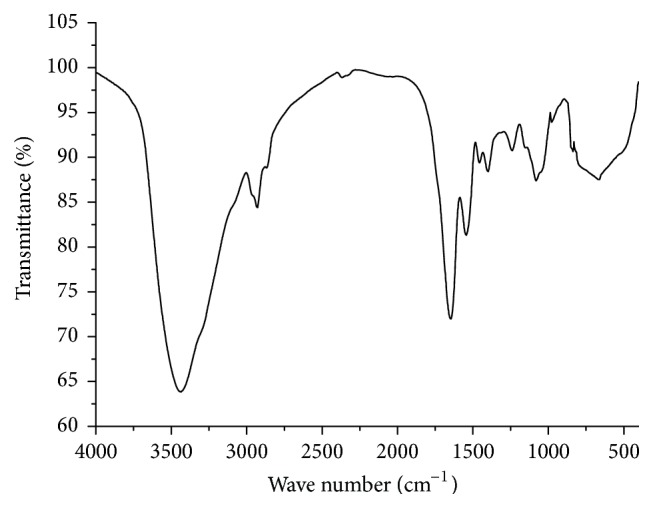
FTIR spectra reveal the chemical constitution of SPMP-2a.

The FTIR spectra of SPMP-2a are shown in Figure 3, and the assignments of most characteristic bands from FTIR are presented in Table 1. An absorption peak at 2927 cm^−1^ indicated the existence of carbohydrate, while absorption peaks at 1645, 1542, and 1398 cm^−1^ suggested the presence of uronic acid in SPMP-2a. The FTIR spectroscopy result showed that SPMP-2a belongs to an acid Se polysaccharide with *α*-D-glucopyranoside bond.

### 3.4. Effects of SPMP-2a and H_2_O_2_ on HaCaT Cell Proliferation

**Figure 4:**
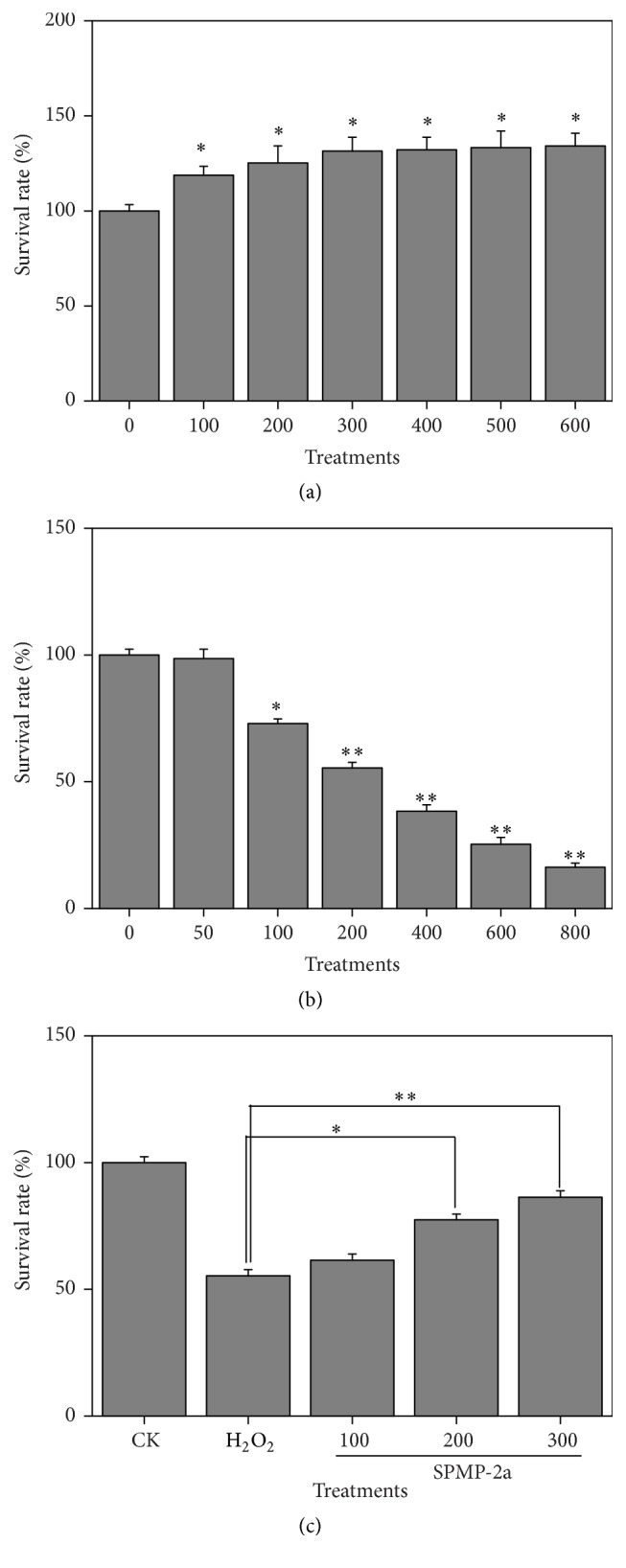
Effects of SPMP-2a and H_2_O_2_ on the survival rate of HaCaT cells. Effects of SPMP-2a (a) and H_2_O_2_ (b) on the HaCaT cell survival rate, as well as protective effects of SPMP-2a against oxidative damage induced by H_2_O_2_ (c). “*∗*” and “*∗∗*” in the histogram indicate significant difference at *α* = 0.05 and 0.01, comparing with the control group (a and b) and model group (c), respectively.

The effect of SPMP-2a on HaCaT cell proliferation is shown in Figure 4(a). Different SPMP-2a concentrations significantly promoted HaCaT cell growth depending on the dosage compared with the control group. SPMP-2a was nontoxic to HaCaT cells. However, the proproliferation effect of SPMP-2a on HaCaT cell growth did not show further increase when the concentration was higher than 300 *μ*g/mL. Therefore, three different doses of SPMP-2a (100, 200, and 300 *μ*g/mL) were used to study the protective effect of SPMP-2a against H_2_O_2_-induced oxidative damage. H_2_O_2_ was used to treat the HaCaT cells, and the effects of H_2_O_2_ on HaCaT cell viability were examined to establish a proper model of oxidative damage in HaCaT cells. The results are shown in Figure 4(b). Increased concentrations of H_2_O_2_ in the 0–800 *μ*mol/L range gradually decreased HaCaT cell viability. H_2_O_2_ concentration higher than 100 *μ*mol/L significantly decreased the cell survival rate. The cell survival rate was only 55.5% after 200 *μ*mol/L of H_2_O_2_ treatment. Cells were damaged, but no cell death took place. As such, 200 *μ*mol/L H_2_O_2_ was selected as the treatment for 6 h to induce cell injury.

### 3.5. Protective Effects of SPMP-2a against H_2_O_2_-Induced Oxidative Damage

The protective effects of SPMP-2a against induced oxidative damage are shown in Figure 4(c). H_2_O_2_ inhibited HaCaT cell proliferation, whereas SPMP-2a significantly reversed the antiproliferation effect of H_2_O_2_ on HaCaT cells. In the preset concentration range, SPMP-2a treatment significantly increased the survival rate of H_2_O_2_-treated HaCaT cells, which was dependent on SPMP-2a concentrations.

### 3.6. Effects of SPMP-2a on Cell Morphological and Apoptosis of the HaCaT Cells

**Figure 5:**
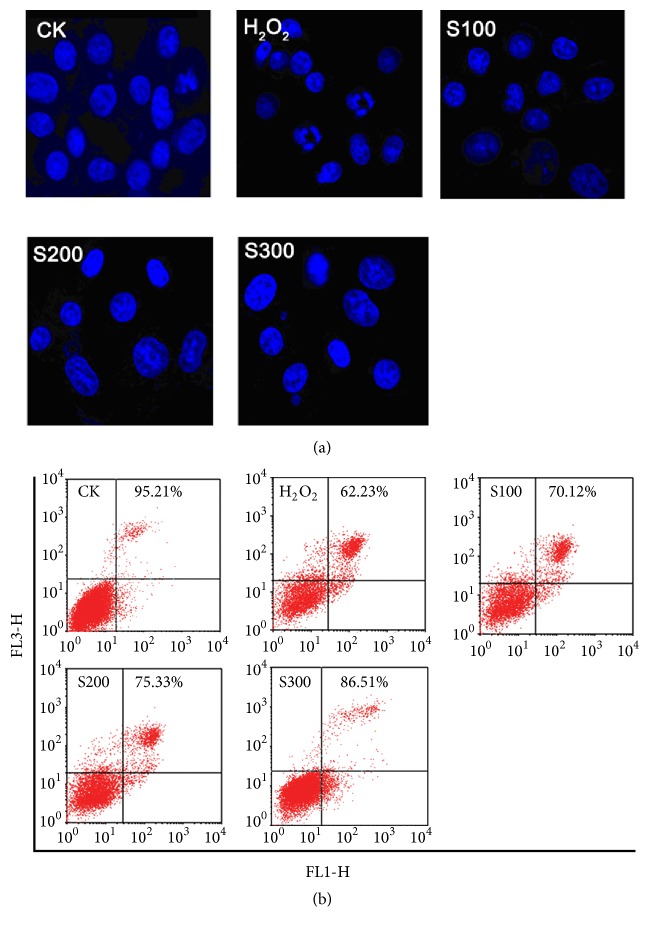
Effects of SPMP-2a on morphological and apoptosis of HaCaT cells. (a) Morphological changes of HaCaT cells revealed by Hoechst 33342 staining. (b) Flow cytometry revealed the apoptosis of H_2_O_2_-treated HaCaT cells. The maximum excitation wavelength is 350 nm. Scale bar = 30 *μ*m.

Confocal laser scanning microscopy images of the Hoechst 33342-stained HaCaT cells are shown in Figure 5(a). Cell morphology in the control group was round with uniform light staining, but the cells in the model group (H_2_O_2_ treatment group) exhibited a blebbing periphery, nuclear condensation, dense granular fluorescence, cell shrinkage, hyperchromatic nuclei, nuclear fragmentation, and apoptotic bodies. The HaCaT cells had better cell morphology (regular round or oval) after the SPMP-2a treatment than the model group. In 100–300 *μ*g/mL concentration range, as the SPMP-2a concentration increased, cell morphology gradually became round and intact with a reduction of apoptotic bodies.

**Figure 6:**
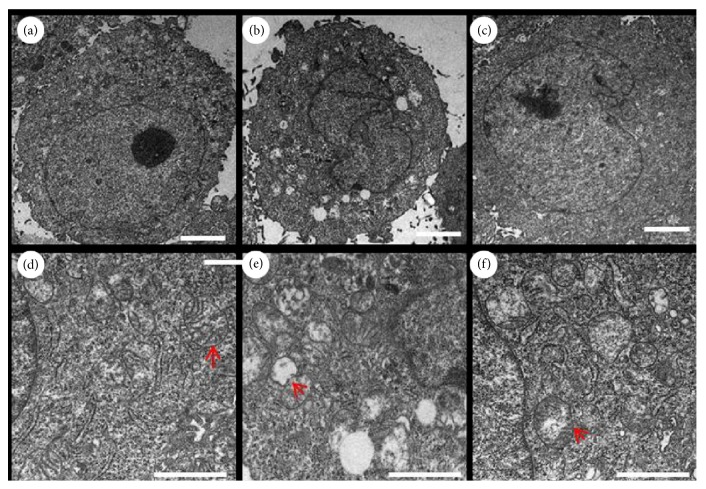
The effect of SPMP-2a on the ultrastructural changes of HaCaT cells exposed to H_2_O_2_. Transmission electron micrograms showed the ultrastructure of HaCaT cells before (a, d) and after H_2_O_2_ injury (b, e) or treated with 300 *μ*mol/L SPMP-2a after the injury (c, f). Red arrows indicated the structure of mitochondria in the cytoplasm of HaCaT cells. Scale bar = 1 *μ*m.

The ratio of live cells and apoptotic cells was determined by Annexin V-FITC/PI staining. The H_2_O_2_-treated HaCaT cells had impaired cell viability, thereby indicating fewer live cells and more apoptotic cells compared with the control group. The SPMP-2a treatment reversed H_2_O_2_ effects on the HaCaT cells as the survival rate of cells increased. The ratios of live cells were 70.12%, 75.33%, and 86.51% after 100, 200, and 300 *μ*mol/L SPMP-2a pretreatments, respectively (Figure 5(b)). Furthermore, numerous round or oval mitochondria were observed in the cytoplasm, and the nuclear membrane was clearly visible in the control groups. In the H_2_O_2_-treated cells, the nuclear membrane was irregular and broken (Figures 6(a) and 6(d)). In addition, swollen and vacuolar mitochondria within cytoplasm were numerous, and the mitochondria displayed ruptured or absent cristae (Figures 6(b) and 6(e)). By contrast, incubation with 300 *μ*mol/L SPMP-2a for 48 h remarkably alleviated all these pathological changes in the H_2_O_2_-treated cells. As shown in Figures 6(c) and 6(f), the amount of swollen and vacuolar mitochondria was significantly lower than that in the H_2_O_2_-treated groups.

### 3.7. Effects of SPMP-2a on the Activities of SOD, CAT, and ROS Content in H_2_O_2_-Treated HaCaT Cells

SOD and CAT activity in the model group (H_2_O_2_ treated group) significantly decreased, whereas ROS significantly increased (*P* < 0.05), thereby indicating that the cell modeling was successful. The H_2_O_2_ treatment led to the reduction of intracellular SOD, CAT, and other antioxidant enzyme activities. The treatment also caused dysfunction of the intracellular endogenous antioxidant enzyme system and accelerated oxidative damage. By contrast, pretreatment with different concentrations of SPMP-2a (100, 200, and 300 *μ*mol/L) for 20 h increased the intracellular SOD, CAT, and other antioxidant enzyme activities, which cleared ROS because of H_2_O_2_-induced cell injury. In the HaCaT cells that were treated with low, medium, and high doses of SPMP-2a, the SOD activity and CAT activity significantly increased, whereas ROS levels declined (Table 2).

### 3.8. SPMP-2a Regulated Bcl-2 Protein Expression in H_2_O_2_-Treated HaCaT Cells

**Figure 7:**
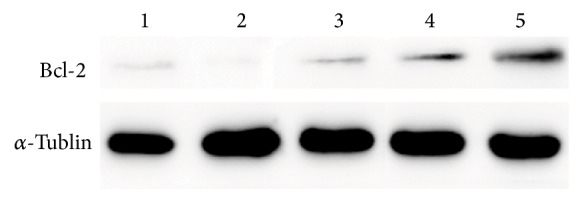
Western blotting revealed SPMP-2a regulated Bcl-2 protein expression in H_2_O_2_-treated HaCaT cells. Lane 1, control group (CK). Lane 2, model group (H_2_O_2_). Lane 3, SPMP-2a 100 *μ*g/mL. Lane 4, SPMP-2a 200 *μ*g/mL. Lane 5, SPMP-2a 300 *μ*g/mL.

The Bcl-2 gene is an important apoptosis suppressor gene. To determine whether SPMP-2a inhibited apoptosis by promoting the expression of Bcl-2, Western blot analysis was performed to detect the expression levels of the Bcl-2 protein and to determine whether SPMP-2a inhibited apoptosis by promoting the expression of Bcl-2.  Figure 7 showed that Bcl-2 protein levels in the model cells were lower than those of the control group. Bcl-2 protein expression levels increased when SPMP-2a concentrations increased, which indicated that SPMP-2a promoted Bcl-2 gene expression and inhibited H_2_O_2_-induced apoptosis.

## 4. Discussion

H_2_O_2_ can be converted into hydroxyl radicals in the presence of intracellular Fe^2+^ through the Fenton reaction [27], which damages intracellular biological macromolecules, DNA, proteins, and lipids, and accelerates oxidative damage and leads to cell death [28]. Therefore, H_2_O_2_ is often used for in vitro modeling of oxidative stress. Studies found that polysaccharide protected cells from H_2_O_2_-induced oxidative damage and prevented cell apoptosis in various species. For example,* Cordyceps* polysaccharide (CPS), a major antioxidative component of* Cordyceps militaris*, significantly protected HL-7702 cells against H_2_O_2_-induced mitochondrial dysfunction by decreasing ROS production [29]. In addition, sulfated polysaccharides* Paliurus* improved the viability of H_2_O_2_-treated RAW264.7 cells, inhibited lipid peroxidation, reduced malondialdehyde levels, increased SOD activity, and protected against H_2_O_2_-induced oxidative stress [30].

Se polysaccharide has both Se and polysaccharide characteristics but with better biological activities [31]. Se polysaccharide gained popularity because of its anticancer, antioxidation, and immunity-boosting activities [32]. In the present study, Se-enriched* P. geesteranus* mycelium was obtained by Se-enrichment fermentation. Mycelium was extracted and purified to produce SPMP-2a. Infrared spectroscopy revealed that SPMP-2a was a homogeneous acidic Se polysaccharide with *α*-D-glucopyranoside bond with a MW of 3.32 × 10^4^ Da. The total sugar and Se content in SPMP-2a were 88.92% and 9.56 mg/g, respectively. In the present study, the model group treated by different SPMP-2a concentrations restored cell morphology and reduced apoptosis rate. Furthermore, within the concentration limits of our study, the concentrations of SPMP-2a increased, and their protective effects on cells against oxidative damage increased proportionally, which indicated that SPMP-2a inhibited H_2_O_2_-induced apoptosis of HaCaT cells and improved cellular antioxidant capacity.

Bcl-2 was the first discovered antiapoptotic gene and is commonly present in the mitochondria, nuclear membranes, and endoplasmic reticulum membranes [33, 34]. The Bcl-2 family is important in the cellular apoptosis process [35]. Overexpression of Bcl-2 protein inhibited TNF-related apoptosis-inducing ligand- (TRAIL-) induced apoptosis by preventing caspase and X-linked inhibitor of apoptosis protein (XIAP) cleavage [36]. Similarly, Bcl-2 protein expression reduced apoptosis of retinal photoreceptor cells and alleviated visual impairment caused by genetic factors and environmental stimuli [37]. In the present study, Bcl-2 protein expression decreased in the H_2_O_2_-induced HaCaT cell damage model, while SPMP-2a treatment increased Bcl-2 protein expression, which was consistent with decreased apoptosis. As such, SPMP-2a promoted Bcl-2 protein expression and regulated H_2_O_2_-induced apoptosis of the HaCaT cell.

Normally, endogenous antioxidant enzymes such as SOD and CAT effectively remove ROS in cells and protect cells from ROS damage. However, SOD and CAT were not sufficient to remove large amounts of ROS in the presence of H_2_O_2_ and other oxidative stress, damaging a number of biological macromolecules within the cells, activating the apoptosis signal transduction pathways, and inducing apoptosis [38]. Li et al. (2004) found that ROS inhibited Bcl-2 protein activity by reducing its phosphorylation and ubiquitination levels and inducing apoptosis [39]. In the present study, the SPMP-2a pretreatment improved SOD and CAT enzyme activity in the damaged HaCaT cells, reduced ROS content, prevented cellular oxidative stress, and significantly increased the survival rates of damaged cells. We speculated that SPMP-2a suppressed HaCaT cell apoptosis by promoting antioxidant enzymes SOD and CAT activities and decreasing intracellular ROS content to reverse Bcl-2 protein level reduction.

## 5. Conclusions

The present study prepared the selenium-combining polysaccharide (SPMP-2a) from* P. geesteranus*. Results show that SPMP-2a is a white flocculent polysaccharide and soluble in water, with a molecular weight of 3.32 × 10^4^ Da and belongs to an acid Se polysaccharide with *α*-D-glucopyranoside bond. Reduced cell viability and elevated apoptotic rates in H_2_O_2_-treated HaCaT cells were observed. However, with the addition of SPMP-2a, cell viability improved, nuclear condensation declined, and cell apoptotic rates dropped significantly. Treatments with SPMP-2a reduced the number of swollen and vacuolar mitochondria in the H_2_O_2_-treated cells compared with the controls. In addition, SPMP-2a increased the SOD and CAT activities and reduced ROS content. Furthermore, SPMP-2a treatment effectively increased Bcl-2 gene expression. Therefore, we concluded that SPMP-2a could improve cellular antioxidant enzyme activities, reduce ROS levels, and increase Bcl-2 gene expression levels, thereby reducing cell apoptosis and protecting HaCaT cells from H_2_O_2_-induced oxidative damage.

## Figures and Tables

**Table 1:** Assignment of the most important infrared bands in SPMP-2a.

Wave number/cm^−1^	Assignments^*∗*^
3434	O-H (stretching)
2927	C-H (stretching)
1645, 1542, 1398	C-O (bending vibrations of uronic acids)
1454, 1238	C-H (deformation)
979	pyranose ring
836	*α*-D-Glucopyranoside bond vibration
665	Se-O-C (asymmetric stretching)

^*∗*^Refer to [24, 25].

**Table 2:** Effects of SPMP-2a on SOD and CAT activities and ROS content of HaCaT cells (*n* = 5).

Group	SOD (U/mg protein)	CAT (U/mg protein)	ROS (% of control)
Control group (CK)	15.47 ± 1.70	5.70 ± 0.38	100.0 ± 1.07
Model group (H_2_O_2_)	5.86 ± 0.56	1.68 ± 0.13	185.1 ± 2.31
SPMP-2a 100 *μ*g/mL	10.24 ± 1.24^*∗*^	1.88 ± 0.24	156.2 ± 4.86
SPMP-2a 200 *μ*g/mL	10.89 ± 1.12^*∗*^	3.05 ± 0.18^*∗*^	138.6 ± 1.63^*∗*^
SPMP-2a 300 *μ*g/mL	12.98 ± 0.32^*∗*^	4.21 ± 0.22^*∗*^	112.5 ± 3.94^*∗*^

^*∗*^Significant difference at *α* = 0.05. *n* = 5 replicates.
